# Protective Effects and Molecular Signaling of n-3 Fatty Acids on Oxidative Stress and Inflammation in Retinal Diseases

**DOI:** 10.3390/antiox9100920

**Published:** 2020-09-26

**Authors:** Ayana Suzumura, Ryo Terao, Hiroki Kaneko

**Affiliations:** 1 Department of Ophthalmology, Nagoya University Graduate School of Medicine, Nagoya 466-8550, Japan; a.suzumura@med.nagoya-u.ac.jp; 2 Department of Ophthalmology, Graduate School of Medicine, The University of Tokyo, Tokyo 113-8655, Japan; rterao-tky@umin.ac.jp

**Keywords:** oxidative stress, n-3 fatty acid, eicosapentaenoic acid, docosahexaenoic acid, retinopathy of prematurity, diabetic retinopathy, age-related macular degeneration

## Abstract

Oxidative stress and inflammation play crucial roles in the development and progression of retinal diseases. Retinal damage by various etiologies can result in retinopathy of prematurity (ROP), diabetic retinopathy (DR), and age-related macular degeneration (AMD). n-3 fatty acids are essential fatty acids and are necessary for homeostasis. They are important retinal membrane components and are involved in energy storage. n-3 fatty acids also have antioxidant and anti-inflammatory properties, and their suppressive effects against ROP, DR, and AMD have been previously evaluated. α-linolenic acid (ALA), eicosapentaenoic acid (EPA), docosahexaenoic acid (DHA), and their metabolites have been shown to alleviate retinal oxidative stress and inflammation involving various biological signaling pathways. In this review, we summarize the current understanding of the n-3 fatty acids effects on the mechanisms of these retinal diseases and how they exert their therapeutic effects, focusing on ALA, EPA, DHA, and their metabolites. This knowledge may provide new remedial strategies for n-3 fatty acids in the prevention and treatment of retinal diseases associated with oxidative stress and inflammation.

## 1. Introduction

Oxidative stress is defined as a disturbance of the balance between the production of free radicals and reactive metabolites: reactive oxygen species (ROS) and the protective mechanism by antioxidants, respectively. The products of oxidation or nitrosylation by ROS reduce biological activity and lead to the loss of energy metabolism, cell signaling, transport, and other key cellular functions. These altered products also target proteasome degradation, further reducing cellular function [1]. In addition, ROS are important signaling molecules that play an essential role in the progression of inflammation [2]. Oxidative stress induces the production of oxidized proteins and glycated products as well as lipid peroxidation, and results in an inflammatory response. In inflammation, hypoxia-inducible factor (HIF) is stabilized, which induces the sequential growth of blood vessels and enables the downstream transcription of angiogenic factors, including vascular endothelial growth factor (VEGF), activation of nuclear factor-kappa B (NF-κB), augmentation of cyclooxygenase (COX)-2 expression, and increased levels of proinflammatory cytokines, like tumor necrosis factor α (TNF-α) and interleukin (IL)-6 [3,4,5,6,7]. Therefore, ROS are involved in a wide spectrum of diseases, including Alzheimer’s disease, Parkinson’s disease, cancer, and diabetes mellitus [8,9,10,11,12,13]. Among ocular tissues, the retina has the highest oxygen consumption per gram of tissue in the human body and requires large amounts of adenosine triphosphate to support cellular functions. However, because of the high metabolism, the retina is vulnerable to oxidative stress damage [14]. For this reason, the retina can be a major site of ROS production and, thus, ROS have been reportedly involved in a variety of retinal diseases, including retinopathy of prematurity (ROP) [15], diabetic retinopathy (DR) [16], and age-related macular degeneration (AMD) [17]. However, the roles of oxidative stress in these disorders remain elusive and a therapeutic strategy has not been established.

Lipids provide energy storage and serve as structural components of cell membranes, ensuring the homeostasis of these barriers. Lipids can also act as signaling molecules, influencing many processes, including gene expression [18,19]. Long-chain n-3 (also called omega-3) fatty acids are particularly essential for the normal growth and neural development of the brain and eye [20,21]. Human bodies cannot efficiently synthesize n-3 fatty acids and, therefore, we need to consume foods that provide adequate amounts of n-3 fatty acids, including fish and fish oil products [22,23,24]. Several randomized controlled trials have shown that oral consumption of n-3 fatty acids is effective in improving inflammation, cardiovascular disease, and peripheral arterial disease [25,26,27,28]. Furthermore, n-3 fatty acids supplementation was recently shown to be beneficial in suppressing ocular diseases, possibly via antioxidant and anti-inflammatory effects [29].

In this review, we summarize current knowledge regarding the therapeutic effects of n-3 fatty acids and their mechanisms, focusing on the pathogenesis of retinal diseases associated with oxidative stress and inflammation.

## 2. The Metabolism of n-3 Fatty Acids and Their Suppressive Effects against Oxidative Stress and Inflammation

Eicosanoids generation by COX, lipoxygenase, and cytochrome P450 enzymes from arachidonic acid (AA) indicate that the eicosanoids can induce oxidative stress, inflammation, and vascular function as lipid mediators [30]. Although AA is also an essential component of cell membranes and plays an integral role in growth during fetal development [31], n-6 fatty acids, including AA, have been strongly linked to inflammation [32]. By contrast, n-3 fatty acids suppress oxidative stress and inflammation, and could even inhibit the generation of AA-derived eicosanoids [33].

Biological roles of n-3 fatty acids have been investigated with a focus on α-linolenic acid (ALA), docosahexaenoic acid (DHA), and eicosapentaenoic acid (EPA). Their multiple roles in homeostasis and suppressive effects on various diseases have been elucidated by recent studies [34,35,36]. DHA, one of the n-3 fatty acids, accounts for approximately 20% of the retinal weight and affects both the survival and development of neurons and retinal vascular cells [37,38,39,40]. EPA, one of the major dietary n-3 fatty acids, is present in the blood. Only a small amount of EPA is stored in human tissues, because EPA is rapidly metabolized in the biosynthesis of DHA and eicosanoids. Previous reports have implied that EPA suppresses AA-derived eicosanoids, which is associated with retinal neovascular abnormalities, vascular permeability, and inflammation [29]. ALA is present in vegetable oils like canola and soybean oil, nuts, and some green leafy vegetables, and is an essential component of human diets. Only a small amount of EPA and DHA can be synthesized from ALA by the human body because there are large amounts of n-6 fatty acids in the diet, which compete for the same enzymes [41,42,43]. DHA and EPA are produced by algae and other aquatic plants and are abundant in fish. Because it is difficult to obtain an adequate DHA and EPA intake through diet alone [44], DHA and EPA supplementation rather than of ALA is recommended [45]. 

**Figure 1:**
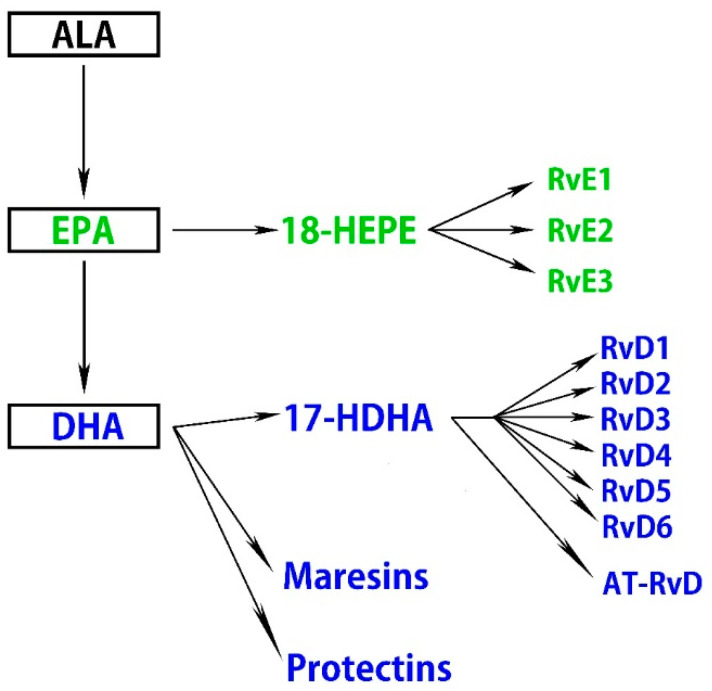
n-3 fatty acids are metabolized to specialized proresolving mediators (SPMs).

A high intake of n-3 fatty acids ameliorates retinal pigmented epithelial (RPE) and photoreceptor degeneration and N-retinyl-N-retinylidene ethanolamine (A2E) accumulation, partly by reducing the production of inflammatory eicosanoids, including prostaglandin E2 (PGE2) and leukotriene B4 (LTB4) [46]. Moreover, n-3 fatty acids attenuate ischemia-induced COX-2 and HIF expression, associated with alleviating neuronal damage [47]. ALA suppresses ROS production, chelates metal ions, inhibits NF-κB activation independent of its antioxidant function, and reduces the oxidized forms of other antioxidants, including vitamin C, vitamin E, and glutathione [48]. Some therapeutic effects of oral ALA administration have been reported, and it may have health benefits independent of its metabolites. ALA also exhibits retinal neuroprotective effects against oxidative stress and nuclear factor (erythroid-derived 2)-like 2 (Nrf2) signaling in retinal neurons [49]. DHA and EPA downregulate insulin-like growth factor-1 (IGF-1), attenuate the activation of NF-κB, IL-1β, VEGF, and TNF-α, and suppress inflammation in cytokine-stimulated human retinal vascular endothelial cells [50,51,52,53]. Several lines of evidence indicate that DHA and EPA improve nitric oxide (NO) bioavailability and decrease ROS production, which correlates with the suppression of VEGF-mediated angiogenic signaling and Nrf2 activation in a dose-dependent manner [54,55,56]. Another study demonstrated that DHA protects retinal photoreceptors from oxidative stress-induced apoptosis [57]. Furthermore, these anti-inflammatory effects of DHA are partly accomplished by downregulating sphingomyelinases; enzymes that catalyze the hydrolysis of sphingomyelin to proinflammatory ceramide [58]. Metabolites of n-3 fatty acids in addition to DHA and EPA also have bioactivity. Specialized proresolving mediators (SPMs) are part of a larger group of proresolving molecules. SPMs include maresins, protectins, and resolvins metabolized from DHA and EPA via 17-hydroxy docosahexaenoic acid (17-HDHA) and 18-hydroxyeicosapentaenoic acid (18-HEPE), respectively [59] (Figure 1). 

It was previously confirmed in in vitro experiments that 17-HDHA and 18-HEPE inhibit TNF-α formation in the macrophage [60]. Maresin 1 (7R,14S-dihydroxy-docosa-4Z,8E,10E,12Z,16Z,19Z-hexaenoic acid), a recently identified lipid mediator generated endogenously by macrophage enzymes, is synthesized from DHA. It can regulate proinflammatory cytokines, including NF-κB, IL-1β, IL-6, and TNF-α [61]. Resolvins are produced from DHA and EPA and are denoted as the D-series (RvD1–RvD6) and E-series (RvE1–RvE3), respectively, while certain isomers of RvDs termed aspirin-triggered resolvin Ds (AT-RvDs) require acetylated COX-2 in the presence of aspirin or other nonsteroid anti-inflammatory drugs for their synthesis [62]. Resolvins exhibit their proresolving effects through specific G-protein coupled receptors (GPR). Currently, several receptors have been identified that recognize members of the D- and E-series of resolvins, including, D resolvin receptor 1 (DRV1)/GPR32, lipoxin A4 receptor/formyl peptide receptor 2, DRV2/GPR18, resolvin E1 receptor/ChemR23, and leukotriene B4 receptor 1 (BLT1) [63,64]. 

RvD1 inhibits ROS generation and suppresses oxidative stress-induced apoptosis of macrophages through the repression of NAPDH oxidase (NOX)2 activation and upregulation of anti-apoptotic protein expression [65]. RvD1 and RvD2 alleviate the expression of inflammatory cytokines, including TNF-α, IL-1α, IL-1β, CC-chemokine ligand 2 (CCL2), and IL-6, in addition to NF-κB and activator protein-1 [66]. AT-RvD1 reduces oxidative stress, lipid peroxidation, apoptosis, and NF-κB signaling in mice after hyperoxia [67]. It also displays an antioxidant effect via Nrf2 elevation [68]. RvE antagonizes BLT1; a proinflammatory receptor for LTB4 expressed in ocular macrophages and retinal glial and endothelial cells [69,70]. Furthermore, because BLT1 regulates ROS release and apoptosis, RvE1 might contribute to the inhibition of apoptosis as an antagonist of BLT1 [70]. Indeed, RvE1 modulates ROS generation by suppressing NOX2 activation [71]. It also attenuates proinflammatory signals, including NF-kB activation, and enhances phagocytosis [72]. Additionally, RvE1 serves as an agonist for ChemR23, which is present on the surface of retinal microglia, regulating TNF-α production as one of the potent regulators of angiogenesis [73,74].

**Figure 2:**
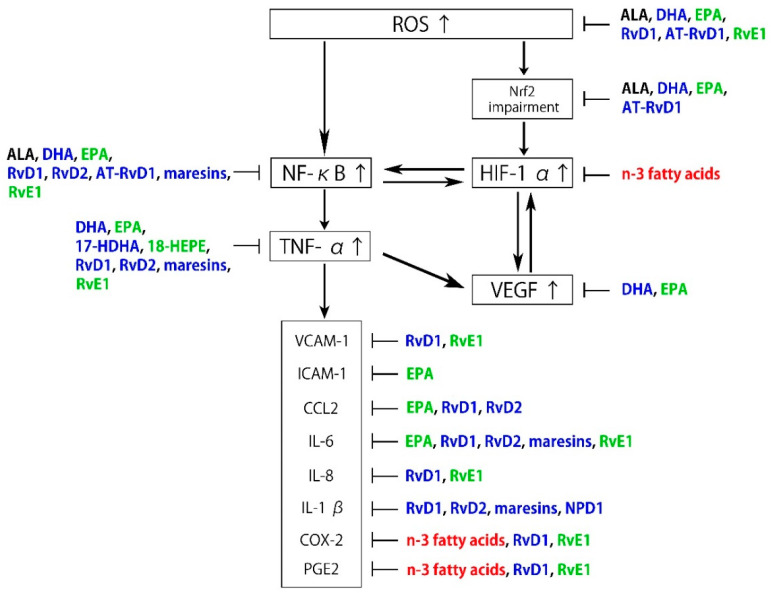
Schematic representation of the inhibitory effects of n-3 fatty acids and specialized proresolving mediator (SPM) on reactive oxygen species (ROS) and inflammation. Black: α-linolenic acid (ALA), Blue: docosahexaenoic acid (DHA) and its metabolites, Green: eicosapentaenoic acid (EPA) and its metabolites, Red: n-3 fatty acids.

Neuroprotectin D1 (NPD1) is biosynthesized from DHA and also exhibits anti-inflammatory and neuroprotective activities [75], partly by interacting with GPR32 [76]. It has been proposed that NPD1 protects RPE cells from oxidative stress-induced apoptosis by inducing the phosphatidylinositol 3-kinase/Akt pathway and inhibiting IL-1β-stimulated COX-2 expression [77,78,79]. NPD1 synthesized in RPE cells also protects photoreceptors and retinal ganglion cells (RGCs) [80,81]. As described above, n-3 fatty acids and their metabolites are associated with the regulation of the pathogenesis of ROS and inflammation (Figure 2). 

## 3. Pathology of Retinopathy of Prematurity (ROP) and Its Relationship to n-3 Fatty Acids

### 3.1. Pathogenesis of Retinopathy of Prematurity (ROP)

ROP is a retinal vascular disease leading to visual impairment and blindness in premature infants. Globally, approximately 190,000 preterm newborns are affected, 20,000 of whom become severely visually impaired or blind from ROP annually [82]. In the first stage of ROP, the normal retinal blood vessels that would grow in the womb are disrupted and a relative peroxidation state occurs when a premature infant is exposed to high oxygen in the incubator after birth [83]. The postnatal hyperoxic environment for premature neonates induces severe growth attenuation and vasodilation in tissues that have not yet fully developed; it reduces VEGF expression partly due to hyperoxia-induced HIF downregulation [84,85]. It is widely accepted that the use of supplemental oxygen, oxygen concentration and duration, and prolonged mechanical ventilation are risk factors, which contribute to ROP severity. Downregulated VEGF regresses the developing retinal vessels. The relative hypoxia that occurs as the infant is returned to normoxia after discontinuation of oxygen therapy worsens as the infant grows, resulting in hypoxic retinal damage. The neonatal retina becomes relatively hypoxic, leading to excessive VEGF production via HIF signaling [85,86,87]. Although HIF/VEGF signaling is particularly essential for fetal growth and vascular development under physiological hypoxia condition, excessive and/or ectopic HIF/VEGF causes retinal neovascularization sprouting towards the vitreous cavity. Retinal neovascularization causes tractional retinal detachment and retinal hemorrhage, which can lead to blindness [88]. These proliferative stages of ROP are strongly associated with IGF-1 [89,90]. IGF-1, which is sufficient to allow vessel growth in womb, is not maintained at in womb levels on premature birth, and vascular growth halts. As the premature infant matures, the retina without mature or sufficient vascularization suffers from hypoxia. When the IGF-1 concentration increases sufficiently to allow activation of the VEGF pathways, VEGF-driven endothelial cell proliferation may proceed and induce retinal pathological neovascularization [91]. 

The pathogenesis of ROP involves multiple signaling pathways induced by oxidative stress. NO production in the retina is increased in hypoxia by NF-κB, whereas administration of nitric oxide synthase (NOS) inhibitors and gene deletion of endothelial NOS effectively reduced ROP severity, suggesting that endogenous NO plays an important role in ROP [92,93,94]. Additionally, NOX is involved in retinal angiogenesis through ROS generation [95]. NOX4 is highly expressed in retinal endothelial cells and regulates VEGF-induced ROS in an animal model representative of ROP, enhancing endothelial cell proliferation [96]. Furthermore, the overproduced ROS causes neovascularization in the retina by activating the Janus kinase-signal transducer/activator of the transcription signaling pathway [97,98,99].

In addition to these oxidative stresses, inflammation is a key modulator in the development and progression of ROP [100,101]. Animal studies have proposed that retinal inflammation leads to abnormal retinal vascular development, suggesting that inflammation is involved in ROP pathogenesis [102,103,104]. In addition, recent investigations have demonstrated that the IL-1 family, consisting of both pro- and anti-inflammatory cytokines, is pivotal in ROP pathogenesis, and that increased complement activation induced microglia activation, which leads to increased inflammation [105,106,107]. Additionally, the NF-κB, IL-6, and TNF-α expression levels were significantly elevated in the retinas of oxygen-induced retinopathy (OIR) rats, which is a widely used ROP model [108].

### 3.2. Therapeutic Effects of n-3 Fatty Acids on Retinopathy of Prematurity (ROP)

Numerous investigations using the OIR model have shown that n-3 fatty acids administration during the neovascular phase significantly reduces neovascularization without altering vasodilation or normal vessel regeneration [109]. Their supplementation is a potent modifier of IGF-1 and reduces OIR severity [110]. n-3 fatty acids suppressed TNF-α expression in the OIR model, decreasing the size of the retinal avascular area [74,111]. 

Additionally, DHA administration reduced lipid peroxidation markers in a piglet model of severe hypoxia-reoxygenation, indicating the established benefit of neuroprotection from oxidative stress [112]. Regarding the SPMs, NPD1, RvD1, and RvE1, they presented significant protection from vaso-obliteration and neovascularization through TNF-α expression regulation in OIR model mice [74]. These studies suggest that n-3 fatty acids may contribute directly to protective effects against the molecular pathogenesis of ROP.

n-3 fatty acids are deficient in preterm infants because of their maternal origin [113,114]. Therefore, exogenous administration of n-3 fatty acids is necessary for such infants. In a double-blind parallel clinical trial, preterm infants with a birth weight between 1000 and 1500 g were assessed to define the effect of DHA on ROP. Infants receiving 75 mg DHA/kg/d displayed significantly reduced severe (stage 3) ROP compared with those receiving high oleic sunflower oil, which is rich in n-6 fatty acids [115]. Another randomized double-blinded controlled trial enrolling 160 premature infants with a gestational age of less than 32 weeks and birth weight of <1500 g found that 300 mg/d n-3 fatty acids could lower ROP frequency and severity [116]. Furthermore, n-3 fatty acid administration resulted in less laser treatment against retinal pathological neovascularization, suggesting that n-3 fatty acids may prevent the development of aggressive ROP [117,118]. By contrast, intravenous administration of fish-oil-based lipid emulsion (SmofLipid^®^, Fresenius Kabi), a blend of plant- and animal-based lipid emulsions with a higher n-6/n-3 fatty acids ratio than in the diet, resulted in less ROP but did not alter the requirement for laser treatment or the ROP frequency [119,120]. These conflicting results may stem from the optimal ratio of n-6 fatty acids, such as AA, to n-3 fatty acids for the developmental stage in preterm infants [121]. Recent randomized controlled clinical trials have studied the effect of n-3 fatty acid or a combination of DHA and AA on ROP outcome and may help to solve these problems (ClinicalTrials.gov Identifier: NCT02486042, NCT03201588).

## 4. Pathology of Diabetic Retinopathy (DR) and Its Relationship to n-3 Fatty Acids

### 4.1. Pathogenesis of Diabetic Retinopathy (DR)

Diabetes mellitus is a chronic degenerative disease characterized by hyperglycemia and is one of the most major public health challenges, with a global prevalence approaching 400 million [122]. It is associated with macrovascular and microvascular complications, including coronary artery disease, stroke, neuropathy, nephropathy, and retinopathy [123]. DR is a potentially blinding complication of diabetes and a significant cause globally of visual impairment [124]. The retina because of its anatomical and physiological specialization required for vision is subject to specific constraints compared with other nervous system tissues and may be vulnerable to diabetes mellitus, in which neurotransmitter production is inhibited and a proapoptotic or proinflammatory response is induced [125]. Approximately 33% of diabetic patients display signs of DR and 10% have vision-threatening stages of retinopathy [124,126]. 

Chronic hyperglycemia is a significant risk factor for the long-term progression of DR according to the Wisconsin Epidemiologic Study of Diabetic Retinopathy, the United Kingdom Prospective Diabetes Study, and the Barbados Eye Studies [127,128,129]. However, notably, observational follow-up of the Epidemiology of Diabetes Interventions and Complications study found that hemoglobin A1c levels did not necessarily correlate with the risk for further retinopathy four years after the completion of the Diabetes Control and Complications Trial [130]. Furthermore, recent studies have reported that small glutamyl transpeptidase -binding proteins and “glycemic memory”, which can persist for a long time even after the blood glucose concentration returns to normal, play a crucial role in DR [131,132]. Therefore, the involvement of another pathological factor is implicated in addition to hyperglycemia. 

Recent studies have shown that oxidative stress is an influential factor in diabetic complications [133,134]. Metabolic abnormalities caused by hyperglycemia can lead to excessive ROS production, and ROS accumulation induces oxidative stress, damaging the tissues in and around retinal blood vessels and ultimately leading to DR. NOX2 is the predominant source of cytosolic ROS and the cytosolic ROS signaling is activated in the early stages of diabetes, leading to mitochondrial damage [135,136,137]. Mitochondria are a major source of ROS and mitochondrial ROS overproduction results in four classical mechanisms; the polyol, hexosamine, protein kinase C, and advanced glycation end-product pathways [138,139,140,141,142]. 

The activation of various pathways, including the renin-angiotensin system (RAS), inflammatory pathways mediated by NF-κB and HIF-1, and Nrf2 antioxidant defense system, have been studied and found to be involved in apoptosis and angiogenesis. RAS regulates pathological angiogenesis and inflammation as well as the systemic blood pressure, which is termed “tissue RAS” [143,144]. Recently, the involvement of the (pro)renin receptor ((P)RR) in the activation of the retinal RAS and its intracellular signaling has been elucidated, and it has been suggested that selective (P)RR targeting in particular may be a promising objective for DR therapy [145]. Additionally, angiotensin II (ANG-II) is the key product of RAS and stimulates NOX-derived ROS formation, directly damaging endothelial cells [95,146]. In the diabetic retina, upregulated ANG-II production, which leads to neuronal extracellular-signal-regulated kinase (ERK) activation, results in synaptophysin degradation, suggesting that RAS also directly affects neurons [147]. Regarding NF-κB, recent studies have reported that not only ROS but also ANG-II activates NF-κB, which in turn promotes the expression of VEGF and proinflammatory mediators, including intercellular adhesion molecule-1 (ICAM-1), vascular cell adhesion molecule-1 (VCAM-1), CCL2, and COX-2 [148,149,150]. In addition, several lines of evidence indicate that HIF-1 regulates VEGF expression, which is a crucial factor affecting angiogenesis in DR pathogenesis [151]. Nrf2 initiates the transcription and expression of various downstream detoxification and antioxidant enzymes, which is impaired under the diabetic environment [152]. Recently, it was determined that Nrf2 is expressed in human retina, and diabetic Nrf2-knockout mice displayed aggravated retina-blood barrier dysfunction, leading to the release of inflammatory factors and exacerbated neuronal dysfunction [153]. Therefore, the suppression of ROS production by these pathways as a novel therapeutic strategy has been investigated in several studies.

### 4.2. Therapeutic Effects of n-3 Fatty Acids on Diabetic Retinopathy (DR)

Several studies using streptozotocin (STZ)-induced diabetic rats, which is a widely used diabetic animal model, concluded that ALA supplementation can prevent pericyte loss, ameliorate oxidative stress, normalize NF-κB activation, and reduce VEGF expression in the diabetic retina [154,155,156]. Additionally, STZ-induced diabetic rats have significantly higher IL-6 levels and lower brain-derived neurotrophic factor (BDNF) levels in the serum, both of which are recovered to near normal by ALA administration, indicating that it provides both, anti-inflammatory and neuroprotective effects [157]. Furthermore, ALA inhibits RGCs loss and reduces the thinning of the inner and outer retinal layers in STZ-induced diabetic mice [158]. Various studies demonstrated reduced levels of neurotrophic factors, including BDNF, which are essential for retinal neuronal cell survival in diabetic patients as well as in diabetic animals with increased oxidative stress [159,160,161]. Consistent with these results, intravitreal 18-HEPE administration also induced BDNF upregulation and led to neuroprotection in early-stage DR [162].

DHA also has suppressive effects against DR pathogenesis. The oil mixture of DHA and EPA protected RPE cells against the high glucose-induced reduction of mitochondrial dehydrogenase activity and ROS and TNF-α overproduction [163]. A single EPA administration improved vascular endothelial function in type 2 diabetic Otsuka Long-Evans Tokushima fatty rats by inhibiting ERK, decreasing NF-κB activation, and reducing COX-2 expression [164]. In addition, oral EPA administration alleviated retinal neurodegeneration via BDNF in Müller glia cells in the early DR stages [162]. Clinically, a randomized controlled trial conducted in patients with diabetic macular edema (DME), which was induced by ROS and DR inflammation, found that the combined administration of oral DHA and intravitreal anti-VEGF significantly improved DME compared with a single treatment with anti-VEGF drugs. This anatomical improvement in central retinal thickness was accompanied by an amelioration of visual acuity [165]. Another clinical trial concluded that EPA administration in type 2 diabetic patients increases endogenous antioxidant enzymes, including superoxide dismutase and glutathione peroxidase, and decreases the level of malondialdehyde, a classic biomarker of oxidative stress [166]. 

n-3 fatty acids also displayed DR attenuation, partly by suppressing rod photoreceptor and inner retinal dysfunction in STZ-induced diabetic rats [167]. In the leptin-receptor-deficient (db/db) mouse, which is a major rodent model of type 2 diabetes mellitus, n-3 fatty acids significantly preserved retinal function, whereas retinal function gradually deteriorated in db/db mice on an n-6 fatty-acidsrich diet [168]. Moreover, it was reported that a low tissue n-3/n-6 fatty acids ratio can be a high-risk factor for many diseases, with one previous study showing a decreasing trend in the n-3/n-6 fatty acids ratio in human donor retinas with DR in comparison with age-matched control retinas [169,170]. Interestingly, the n-3/n-6 fatty acids ratios in retina and serum of Nile rat and Akita mouse, type 2 and type 1 diabetes mellitus animal models, respectively, improved significantly by dietary supplementation of n-3 fatty acids [170]. In a randomized controlled trial, a six-year follow-up analysis in individuals with type 2 diabetes mellitus elucidated that a dietary intake of a minimum of 500 mg/d n-3 fatty acids could reduce the risk for developing DR by 48% [171]. The results from a cross-sectional study evaluating multiethnic Asian adults with type 2 diabetes mellitus showed that higher fish consumption, and thus n-3 fatty acids intake, was significantly associated with reduced odds for severe DR and was correlated with wider retinal vascular caliber in diabetes mellitus patients without retinopathy [172]. Given previous reports describing that narrower retinal vascular diameters are associated with an increased risk for DR, n-3 fatty acids may be effective not only in halting DR progression but also in preventing DR preclinically [173,174].

Maresin 1 can inhibit ROS generation induced by high glucose in a dose-dependent manner [175,176,177]. In addition, maresin-like mediators (14,22-dihydroxy-docosa-4Z, 7Z, 10Z, 12E, 16Z, 19Z-hexaenoic acids) ameliorated impaired macrophage function by high glucose, suppressing the chronic inflammation in diabetic wounds [176]. RvD1 also decreased NF-κB levels in photoreceptors stimulated with high glucose and modified VEGF content in exosomes released by photoreceptors [178]. Additionally, BLT1 expression was increased in diabetic mice retina and retinal glial cells exposed to high glucose also demonstrated enhanced BLT1 expression, whose antagonist RvE1 attenuated its ROS pathway and apoptosis signaling [70,179]. It was also elucidated that intravitreal RvD1 injection suppresses NF-κB activation and downregulates the retinal IL-1β levels of the STZ-induced diabetic rats [180].

## 5. Pathology of Age-Related Macular Degeneration (AMD) and Its Relationship to n-3 Fatty Acids

### 5.1. Pathogenesis of Age-Related Macular Degeneration

AMD is the leading cause of irreversible blindness in patients aged over 50 years in developed countries, affecting 170 million globally [181]. Because aging is one of the greatest risk factors, the disease prevalence is expected to increase with the aging of society [182]. There are two types of AMD: “dry” and “wet”. Dry AMD is a chronic disease that results in vision loss because of the “geographic atrophic” death of photoreceptors and RPE cells [183]. Wet, or neovascular, AMD can also cause significant vision loss by the formation of choroidal neovascularization (CNV), as a result of pathological angiogenesis [184]. In both types, oxidative stress is strongly implicated in their pathogenesis [185,186]. While an adequate oxygen supply to the retina is necessary to maintain retinal functions, a high supply also induces retinal ROS production. Increased ROS levels and attenuated antioxidant cellular defense systems in RPE cells lead to AMD pathogenesis [187,188,189]. 

These sources of ROS include oxidative loads from cigarette smoking and high-energy light exposure. Several systematic reviews have found smoking to be a major risk factor for AMD [190,191,192]. Additionally, recent in vitro studies have shown that exposure to cigarette smoking induces a dose- and time-dependent increases in endoplasmic reticulum stress markers, enhanced ROS, and apoptosis of RPE cells [193,194,195]. Furthermore, sunlight exposure is a significant risk for AMD [196]. Indeed, RPE cells are particularly susceptible to wavelengths within the blue region of the solar spectrum and blue light is the most energetic radiation reaching the macula, resulting in RPE cell apoptosis and necrosis [197,198,199]. 

Drusen formation is a manifestation of early-stage AMD, but little is known about its origin. Some hypotheses suggest that a significant amount of drusen originates from blood, while others suggest that it is derived from cellular debris processed from photoreceptor outer segments and RPE cells [200]. The photoreceptor is an abundant source of DHA-containing phospholipids, which are highly susceptible to damage by increased ROS and oxidative stress levels [201,202]. Active phagocytosis of photoreceptor outer segments by the RPE cells removes oxidatively damaged photoreceptor discs, but impairment of these functions by aging involve the accumulation of toxic proteins, including lipofuscin and extracellular drusen [203,204,205]. A2E is one of the retinoid components of lipofuscin and is involved in blue light-induced RPE cell apoptosis, inflammatory changes, and inducing VEGF expression [206,207,208,209,210]. Chronic inflammation, a prolonged response that can result in tissue damage when the protective response becomes dysfunctional, is also thought to be a contributory factor to AMD. Among proangiogenic factors and inflammatory cytokines, VEGF, CCL2, HIF, and IL-8 are reportedly crucial factors associated with CNV formation [211]. In CNV formation, VEGF induces the proliferation of vascular endothelial cells and promotes macrophage migration [212]. Macrophages/microglia which infiltrate the CNV regions secrete IL-6 and TNF-α, and the attenuation of macrophage migration by blocking CCL2 and downregulating the HIF-1α/VEGF pathway suppresses the leakage and reduce the area of laser-induced CNV, a widely used animal model of neovascular AMD [213,214]. Surgically removed human CNV membranes also indicated that TNF-α derived from macrophages facilitates pathologic angiogenesis in AMD [215]. A previous study reported elevated CCL2, IL-6, and IL-8 levels in the intraocular sample from neovascular AMD patients, and the levels were related to CNV lesion size [216]. Moreover, levels of ICAM-1 and VCAM-1, which contribute to CNV development by means of strong leukocyte-endothelial interactions and cell migration, were elevated in patients with neovascular AMD [217]. Additionally, NF-κB, an essential regulator of IL-6 and PGE2, and COX-2, whose selective antagonist inhibits subretinal fibrosis, are both further crucial regulators of CNV. [19,218,219,220]. Compelling evidence also suggests that ocular infiltration of a specific type of macrophages (M2 macrophage) via the LTB4-BLT1 signaling pathway is intimately involved in the development of laser-induced CNV [69].

### 5.2. Therapeutic Effects of n-3 Fatty Acids on Age-Related Macular Degeneration (AMD)

EPA application substantially reduced ICAM-1 and CCL2 expression in endothelial cells and VEGF and IL-6 expression in macrophages [221]. EPA-fed mice exhibited prominently decreased ICAM-1, CCL2, VEGF, and IL-6 expression and production in the RPE-choroid, resulting in significant suppression of laser-induced CNV. A previous study using the *Ccl2*(−/−)/*Cx3cr1*(−/−) mouse exhibiting focal deep retinal lesions, abnormal RPE, photoreceptor degeneration, and A2E accumulation, demonstrated that a high n-3 fatty acid diet slowed the progression of AMD-like retinal lesions and reduced retinal TNF-α and IL-6 expression levels [46]. A previous study using the *Ccl2*(−/−) mouse revealed that n-3 fatty acids administration increased EPA and AA in the blood and retina, and decreased retinal NF-κB expression, accompanied by increased outer layer thickness [222].

DHA selectively enhances NPD1 synthesis and release through the apical surface of RPE cells [223]. Additionally, NPD1 reduced the leakage area and vascular endothelial cell volume in a laser-induced mouse model of CNV concomitant with the redistribution and ramification of microglia [224,225]. Regarding other SPMs, RvD1 and RvE1 reduced the expression of IL-8, IL-6, PGE2, COX-2, and VCAM-1 induced in choroid–retinal endothelial cells and leukocytes after inflammatory stimulation [226].

An epidemiological study concluded that high plasma total n-3 fatty acids significantly reduced the probability of developing late AMD by 38% [227]. Given that low n-3/n-6 fatty acids ratios occur in human retinas with macular degenerations and the imbalance of n-3/n-6 fatty acids may be involved in AMD pathogenesis, appropriate oral intake of lipids may contribute to AMD management because the n-3 fatty acids concentration in the retina can be modified by dietary composition [228,229]. A recent prospective clinical trial reported that n-3 fatty acids supplementation significantly lowered intravitreal VEGF-A levels in neovascular AMD patients [230]. A prospective cohort study with a mean follow-up time of 4.6 years showed that higher n-3 fatty acids intake was associated with a lower risk for AMD progression [231]. Another large prospective cohort study demonstrated that regular DHA and EPA intake reduced the 10-year incidence of visually significant AMD by 35–45%, indicating that dietary n-3 fatty acids intake may be advantageous for the primary prevention of AMD [232]. With respect to the protective effect on early AMD, the Blue Mountains Eye Study indicated that the dietary intake of n-3 fatty-acid-rich fish at least once weekly was associated with a reduced risk for developing early-stage AMD [233]. Another large prospective cohort study with a 24–28-year follow-up also indicated that a high DHA or EPA intake was correlated with a 17–40% reduction in the risk for visually significant intermediate AMD [234]. Moreover, in the previous meta-analysis and systematic review evaluating 4202 cases with 128,988 individuals from eight cohort studies, a linear association was revealed between n-3 fatty-acid-rich fish consumption and risk for AMD, and higher fish consumption was found to be associated with a lower risk for both, early and late AMD [235]. These results were consistent with the conclusion of the Age-Related Eye Disease Study (AREDS), which retrospectively evaluated the effect of dietary n-3 fatty acids intake on AMD severity and indicated that higher n-3 fatty acids and fish intake was associated with lower odds for neovascular AMD [236].

Some clinical investigations have failed to verify any impact of n-3 fatty acids on progression to advanced AMD. The AREDS2, a randomized, double-masked, controlled trial, was a follow-up study from AREDS designed to prospectively evaluate the impact of n-3 fatty acids supplementation on AMD progression. Participants enrolled in the study were at high risk for late AMD progression, ranging from bilateral large drusen to large drusen in one eye and late AMD in the fellow eye. DHA and EPA supplementation plus the AREDS formulation (antioxidant vitamins C and E, beta carotene, and zinc) displayed no statistically significant reduction in progression to advanced AMD [237]. Correspondingly, another clinical study also concluded that high DHA and EPA intakes, which may prevent or delay the occurrence of visually significant intermediate AMD, were not associated with a reduction in the risk for advanced AMD [234]. The Nutritional AMD treatment-2 (NAT-2) study, conducted in patients with early lesions of age-related maculopathy in the study eye and neovascular AMD in the fellow eye, indicated that oral DHA and EPA supplementation did not modify the rate of visual acuity changes or the time to onset or incidence of CNV in the study eye over the three-year study period [238]. 

Similarly, the post-hoc subgroup analysis from the NAT-2 study reported that DHA supplementation was not significantly associated with drusen count, total diameter, or total area progression on fundus photographs [239]. The divergence in the results of these studies may be influenced by the difference in an uncontrolled baseline diet or basal nutritional status of the participants. Additional randomized controlled trials with low rates of loss to follow-up and consistently good adherence to treatment regimens are required to examine the benefit of n-3 fatty acids for AMD.

## 6. Future Perspectives

Experimental studies and clinical investigations suggest the therapeutic effects of n-3 fatty acids on pathological stages of ROP, DR, and AMD (Table 1). More detailed investigations to elucidate the complex interaction between oxidative stress/inflammation and lipids are required. A better understanding of the mechanisms of lipids acting on the retina and in retinal disorders may allow the establishment of more effective n-3 fatty acids administration. 

## Figures and Tables

**Table 1:** The therapeutic effects of the different n-3 fatty acids, the models in which they are tested, and the mechanisms targeted.

Animal Model			
Administration	Model	Effect	References
n-3 fatty acids	OIR rat	Elevations in IGF-1 levels	[110]
	Leptin-receptor-deficient (db/db) mouse	Preservation the function of photoreceptors, bipolar cells, and inner retina	[168]
	STZ rat	Suppressing rod photoreceptor, inner retinal dysfunction	[167]
	*Ccl2*(−/−)/*Cx3cr1*(−/−) mouse	Reduction of retinal TNF-α and IL-6 expression levels, slowing the progression of AMD-like retinal lesions	[46]
	*Ccl2(−/−)* mouse	Reduction of retinal NF-κb expression levels, increasing outer layer thickness	[222]
ALA	STZ rat	Prevention of pericyte loss, suppression of oxidative stress, normalization of NF-κb activation and IL-6 expression, reduction of VEGF expression, BDNF upregulation	[154,155,156,157]
	STZ mouse	Inhibition of rgcs loss, reduction of the inner and outer retinal layer thinning	[158]
DHA	Piglet model of severe hypoxia-reoxygenation	Reduction of lipid peroxidation markers	[112]
EPA	Otsuka Long-Evans Tokushima fatty rat	Inhibiting ERK, decreasing NF-κb activation, reducing COX-2 expression	[164]
	STZ rat	BDNF upregulation	[162]
	Laser-induced mouse model of CNV	Suppression of ICAM-1, CCL2, VEGF, and IL-6 expression and production in the RPE-choroid	[221]
NPD1	OIR mouse	TNF-α expression regulation	[74]
	Laser-induced mouse model of CNV	Reducing the leakage area and vascular endothelial cell volume	[224,225]
RvD1	OIR mouse	TNF-α expression regulation	[74]
	STZ rat	Suppression of NF-kb activation, downregulation the retinal IL-1β levels	[180]
RvE1	OIR mouse	TNF-α expression regulation	[74]
18-HEPE	STZ rat	BDNF upregulation	[162]
**Clinical Study**			
**Administration**	**Model**	**Effect**	**References**
n-3 fatty acids	ROP	Lowering of ROP frequency and severity	[116,117,118]
	DR	Reduction of DR incidence and odds for severe DR	[171,172]
	AMD	Lowering of VEGF-A levels, reduction in the risk for both early and late AMD	[230,231,233,235,236]
DHA	ROP	Reduction of stage 3 ROP incidence	[115]
	DR	Improvement in central retinal thickness, amelioration of visual acuity	[165]
	AMD	Primary prevention of AMD, reduction in the risk for visually significant intermediate AMD	[232,234]
EPA	AMD	Primary prevention of AMD, reduction in the risk for visually significant intermediate AMD	[232,234]

Abbreviations: ROP, Retinopathy of prematurity; DR, diabetic retinopathy; AMD, age-related macular degeneration; OIR, oxygen-induced retinopathy; DHA, docosahexaenoic acid; NPD1, neuroprotectin D1; RvD1, resolvin D1; RvE1, resolvin E1; 18-HEPE, 18-hydroxyeicosapentaenoic acid; RGCs, retinal ganglion cells; CNV, choroidal neovascularization, IGF-1, insulin-like growth factor-1; TNF-α, tumor necrosis factor α; IL, interleukin; VEGF, vascular endothelial growth factor; BDNF, brain-derived neurotrophic factor; RGC, retinal ganglion cell; ERK, extracellular-signal-regulated kinase; COX-2, cyclooxygenase-2; ICAM-1, intercellular adhesion molecule-1; CCL2, CC-chemokine ligand 2; RPE, retinal pigmented epithelial.

## Abbreviations

17-HDHA	17-hydroxy docosahexaenoic acid
18-HEPE	18-hydroxyeicosapentaenoic acid
A2E	N-retinyl-N-retinylidene ethanolamine
AA	arachidonic acid
ALA	α-linolenic acid
AMD	age-related macular degeneration
ANG-II	angiotensin II
AREDS	the Age-Related Eye Disease Study
AT-RvD	aspirin-triggered resolvin D
BDNF	brain-derived neurotrophic factor
BLT1	leukotriene B4 receptor 1
CCL2	CC-chemokine ligand 2
CNV	choroidal neovascularization
COX-2	cyclooxygenase-2
db/db	leptin-receptor-deficient
DHA	docosahexaenoic acid
DM	diabetes mellitus
DME	diabetic macular edema
DR	diabetic retinopathy
DRV1	D resolvin receptor 1
EPA	eicosapentaenoic acid
ERK	extracellular-signal-regulated kinase
GPR	G-protein coupled receptors
HIF	hypoxia-inducible factor
ICAM-1	intercellular adhesion molecule-1
IGF-1	insulin-like growth factor-1
IL	interleukin
LTB4	leukotriene B4
NAT-2 study	the Nutritional AMD treatment-2 study
NO	nitric oxide
NOS	nitric oxide synthase
NOX	NAPDH oxidase
NPD1	neuroprotectin D1
Nrf2	nuclear factor (erythroid-derived 2)-like 2
NSAIDs	nonsteroidal anti-inflammatory drugs
OIR	oxygen-induced retinopathy
PGE2	prostaglandin E2
(P)RR	(pro)renin receptor
RAS	renin-angiotensin system
RGC	retinal ganglion cell
ROP	retinopathy of prematurity
ROS	reactive oxygen species
RPE	retinal pigmented epithelial
SPM	specialized proresolving mediator
STZ	streptozotocin
TNF-α	tumor necrosis factor α
VCAM-1	vascular cell adhesion molecule-1
VEGF	vascular endothelial growth factor
